# Post-discharge management following hip fracture - get you back to B4: A parallel group, randomized controlled trial study protocol

**DOI:** 10.1186/1471-2318-11-30

**Published:** 2011-06-09

**Authors:** Wendy L Cook, Karim M Khan, Michelle H Bech, Penelope M Brasher, Roy A Brown, Stirling Bryan, Meghan G Donaldson, Pierre Guy, Heather M Hanson, Cheryl Leia, Erin M Macri, Joanie Sims-Gould, Heather A McKay, Maureen C Ashe

**Affiliations:** 1 Centre for Hip Health and Mobility, Vancouver, Canada; 2 Vancouver Coastal Health Research Institute, Vancouver, Canada; 3 Department of Medicine, University of British Columbia, Vancouver, Canada; 4 Providence Health Care, Vancouver, Canada; 5 Department of Family Practice, University of British Columbia, Vancouver, Canada; 6 School of Human Kinetics, University of British Columbia, Vancouver, Canada; 7 Centre for Clinical Epidemiology and Evaluation, Vancouver, Canada; 8 School of Population and Public Health, University of British Columbia, Vancouver, Canada; 9 Department of Orthopaedics, University of British Columbia, Vancouver, Canada; 10 Vancouver Coastal Health Authority, Vancouver, Canada

## Abstract

**Background:**

Fall-related hip fractures result in significant personal and societal consequences; importantly, up to half of older adults with hip fracture never regain their previous level of mobility. Strategies of follow-up care for older adults after fracture have improved investigation for osteoporosis; but managing bone health alone is not enough. Prevention of fractures requires management of both bone health and falls risk factors (including the contributing role of cognition, balance and continence) to improve outcomes.

**Methods/Design:**

This is a parallel group, pragmatic randomized controlled trial to test the effectiveness of a post-fracture clinic compared with usual care on mobility for older adults following their hospitalization for hip fracture. Participants randomized to the intervention will attend a fracture follow-up clinic where a geriatrician and physiotherapist will assess and manage their mobility and other health issues. Depending on needs identified at the clinical assessment, participants may receive individualized and group-based outpatient physiotherapy, and a home exercise program. Our primary objective is to assess the effectiveness of a novel post-discharge fracture management strategy on the mobility of older adults after hip fracture.

We will enrol 130 older adults (65 years+) who have sustained a hip fracture in the previous three months, and were admitted to hospital from home and are expected to be discharged home. We will exclude older adults who prior to the fracture were: unable to walk 10 meters; diagnosed with dementia and/or significant comorbidities that would preclude their participation in the clinical service.

Eligible participants will be randomly assigned to the Intervention or Usual Care groups by remote allocation. Treatment allocation will be concealed; investigators, measurement team and primary data analysts will be blinded to group allocation. Our primary outcome is mobility, operationalized as the Short Physical Performance Battery at 12 months. Secondary outcomes include frailty, rehospitalizations, falls risk factors, quality of life, as well as physical activity and sedentary behaviour. We will conduct an economic evaluation to determine health related costs in the first year, and a process evaluation to ascertain the acceptance of the program by older adults, as well as clinicians and staff within the clinic.

**Trial registration number:**

ClinicalTrials.gov: NCT01254942

## Background

Hip fractures are serious, life-limiting and costly events for older adults. Each year in Canada there are over 27,000 hip fractures [1], and older adults who sustain a low-trauma hip fracture have higher risk of death and disability. In the first year after the fracture, as many as 20% of people die [2]; and up to half of seniors will not regain their pre-fracture level of mobility [3,4] leaving them at risk for further falls [5] and fracture injuries [6-9]. Therefore, despite advances in surgical and medical management following hip fracture, up to half of hip fracture patients do not regain their independence or return to prefracture functional mobility.

After hip fracture, systematic structured follow-up strategies for older adults [10,11] improve investigation rates for osteoporosis; and fracture liaison services [10-23] after fracture are beneficial. Many previous interventions, however, have focused on bone health management, and to our knowledge only a few studies [18,20] have included patient referral for falls risk assessment. Osteoporosis care can be improved, but as fractures occur because of low bone mass and *falls*, it is important to also address falls risk [24]. It is noteworthy that despite being prescribed osteoporosis medication [11] some patients who attended a fracture liaison service had a subsequent fall-related low trauma fracture. Hence, the need to coordinate both *falls prevention and bone health *initiatives [11].

Ferrucci and colleagues [25] discuss that hip fracture can result in "catastrophic disability" for older adults due to the unanticipated deterioration in functional ability [25] in some activities of daily living and mobility. Decreased mobility, in turn, impacts on the risk for future injury. Importantly, older adults who sustain hip fracture are at an increased risk of future hip fracture [26,27]. A systematic review [8] emphasized that previous falls [28], low bone density [28,29], and mobility impairment [28] are major risk factors for second hip fracture. Approximately 90% of fractures occur as a result of a fall [30] and after hip fracture, up to half of people can fall again within the first 6 months following fracture [5,31]. These data emphasise the importance of a two-pronged approach to reducing hip fractures - addressing falls risk as well as low bone mass [32].

Compelling evidence supports the recommendation for strength and balance exercises for falls risk reduction in vulnerable populations [33-36]. Exercise improves lower limb muscle strength and it can maintain bone mass in post-menopausal women [37-39]. A Cochrane systematic review [40] reported six trials that evaluated exercise interventions for people after hospital discharge following hip fracture. Balance and strength measures improved overall; in particular, Binder and colleagues [41] reported a significant improvement in mobility measures after a six month out-patient physical therapy intervention.

Therefore, the purpose of the current study is to evaluate the clinical and cost effectiveness of a novel post-discharge fracture management strategy for community-dwelling older adults after hip fracture. It is anticipated that initiating an individualized outpatient management program after hospital discharge, which includes falls and fracture risk assessment and management, and an individualized exercise program under the ongoing guidance of a physical therapist, will improve mobility, and reduce disability and falls.

## Methods/design

### Study Aims

The primary aim of this study is to assess the effectiveness of a comprehensive post-fracture clinic with extended rehabilitation for community-dwelling older adults following hip fracture on mobility. Secondary aims include prospectively (i) determining costs associated with hip fracture, and our intervention, and (ii) completing a process evaluation as part of our integrated knowledge translation plan.

### Study Design and Setting

We propose a parallel group, pragmatic [42] single blind randomized controlled trial of two different delivery modes of post hip fracture management- Usual Care alone versus Usual Care plus specialized outpatient fracture follow-up management. This study will take place at two academic teaching hospitals in Vancouver, Canada. Vancouver is the largest city in the province of British Columbia and with an estimated population of 578,000 and 13% of the population are adults aged 65 years+ (2006 Census Data; http://vancouver.ca/commsvcs/planning/census/2006/index.htm). Our clinic is located within the Vancouver Coastal Health Authority (VCHA) catchment area.

#### Ethical Approval

We have obtained ethical approval from the University of British Columbia (UBC) Clinical Research Ethics Board, Vancouver Coastal Health Research Institute Ethics Board, and Providence Health Ethics Board. All participants will provide written informed consent prior to participating in the study (Clinical Trials Registration NCT01254942).

#### Participants

We will include 130 community-dwelling older adults (women and men) aged 65 years+ who, with a recent history (within past 3 months) of hip fracture, were admitted to hospital from home and expected to be discharged home. We will exclude older adults who prior to the fracture were: unable to walk 10 meters; diagnosed with dementia; and/or with significant comorbidities that would preclude their participation in the clinical service.

#### Identification of Eligible Participants

Our recruitment coordinator will identify and enrol patients into the study prior to discharge from hospital, rehabilitation unit and/or from a discharge list of eligible older adults. Identification of potential participants will be facilitated by the clinical staff; in addition, recruitment posters will also be placed on the ward and on the Centre for Hip Health and Mobility website. The recruitment coordinator will provide written and verbal information to all eligible participants.

#### Randomization

**Figure 1:**
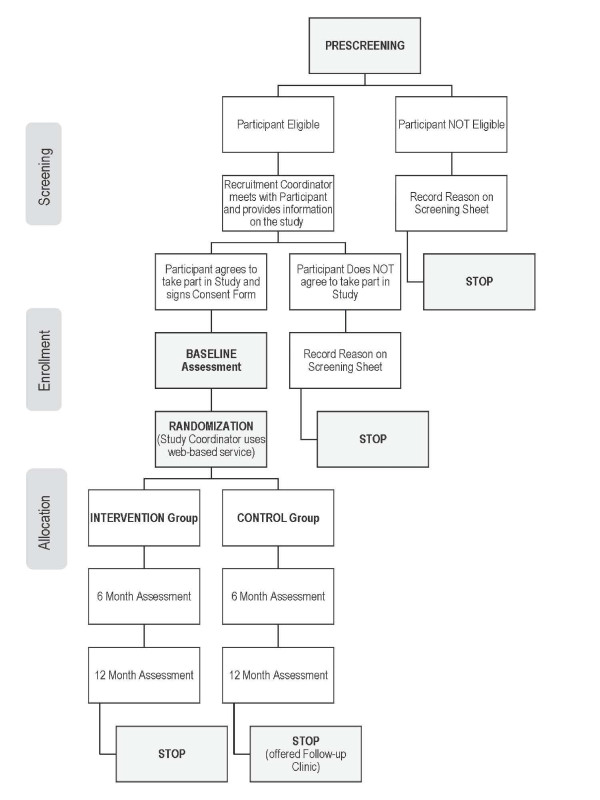
**Flow diagram of the phases of the B4 Clinic trial**.

Treatment allocation will be concealed; a statistician independent of the study will generate the allocation sequence using randomized blocks of varying size; we will stratify randomization by hospital site and sex. This list will be provided to a centralized, web-based randomization service. At the completion of the baseline assessment the study coordinator will use the online service to determine the next allocation. Investigators, the measurement team and data analysts will be blinded to group assignment throughout the trial (Flow Diagram in Figure 1).

#### Study Interventions

##### Usual Care (UC) group

Participants who are randomized to the UC Group will receive usual orthopaedic and rehabilitation post-operative treatment for the hip fracture. Participants in the UC Group will be offered the Fracture Follow-up Clinic at 12 months (please see below for description).

##### Intervention (B4) group

Participants who are randomized to this group will attend an enhanced post-fracture follow-up clinic for outpatient management of falls and fracture risk. The clinic has been designed based on the need to assess both falls risk factors and bone health. A geriatrician will lead the clinic and will provide post-fracture assessment and management. The clinical assessment will focus on four key areas to address secondary prevention of future injury by addressing key domains that contribute to fall and fracture risk, and include assessment of: balance, bone health, brain function and bladder function = B4 Clinic. Based on identified need during the geriatric assessment, participants may also be referred to additional health professionals such as occupational therapist, registered dietician, social worker etc.

In addition, while at the clinic, participants will also be assessed by a physiotherapist who will recommend an exercise program and, may recommend subsequent onsite outpatient physiotherapy visits. Participants will also receive a standardized home exercise program. Following discharge from outpatient physiotherapy, participants will also receive up to four additional telephone calls based on motivational interviewing [43], and we will use techniques to problem solve and facilitate goal setting around mobility and regular engagement in physical activity. If during these sessions, the participant identifies a mobility concern, she/he will be encouraged to follow-up with the B4 Clinic.

### Outcomes

#### Data Collection Protocol

Measurements will be conducted at the Centre for Hip Health and Mobility, Vancouver Coastal Heath Research Institute on the Vancouver General Hospital site or at a participant's home at baseline (3 months post-surgical repair), 6 and 12 months.

#### Primary Outcome

Our primary outcome for this trial is mobility as measured by the Short Physical Performance Battery (SPPB) at 12 months. This valid and reliable measure of mobility has been developed with older adults to assess balance and other key lower extremity functions [44]. The SPPB is composed of three separate tests that are timed and categorized depending on performance: the tests include standing balance, gait speed and sit to stand performance. The SPPB can predict future disability in older adults [45].

### Secondary Health Outcomes

#### Mobility Measures

We will use: (i) the Lower Extremity Measure Scale (LEM) [46], a valid and reliable self-report measure developed specifically for people after hip fracture to assess functional status and to distinguish clinically important change over time [46]; and (ii) the Timed Up and Go (TUG) Test [47] to assess basic mobility function related to transfers, balance and walking capacity. An objective measure of leg strength will be collected.

Falls will be measured by self-reported daily fall diaries, which will be completed and reported monthly to study investigators and followed up by an independent investigator. We will also use the QuickScreen to characterize components of falls risk. This 10 minute test measures key components of falls risk [48] including performance based measures (standing balance, coordination, vision, lower extremity sensation and sit-stands) and self-report information on previous falls and medications. We will assess falls-related self-efficacy by the Short-form Falls Efficacy Scale-International [49].

#### Frailty

We will collect information related to the participants' degree of frailty using two methods: the Clinical Frailty Scale [50], and a frailty index from Fried and colleagues derived from our study measures [51].

#### Patterns of Physical Activity and Sedentary Behaviour

Once a month we will ask participants to complete a Community Healthy Activities Model Program for Seniors (CHAMPS) questionnaire, a valid and reliable 7-day recall self-report tool [52]. We will also ask participants to wear an accelerometer at three time points (baseline, 6 and 12 months) to objectively measure physical activity and sedentary behaviour patterns.

#### Bone Health

We will record the number of best practice bone health options (i) offered and (ii) utilized by all participants, using a list generated from the 2010 Canadian clinical practice guidelines [53] for diagnosis and management of osteoporosis.

#### Cognitive Performance

We will assess global cognition with the Montreal Cognitive Assessment (MoCA) [54] and components of executive function using the Trail Making (Part B) [55] and Stroop Colour Word Tests [56].

#### Continence

Urge incontinence is a risk factor for falls [57,58], and will be ascertained via screening questions [59].

### Descriptive Data

#### Hospital Medical Chart Review

We will collect information regarding previous hospital admissions; participants' health status prior to the hip fracture and at discharge, any complications during hospitalization for the hip fracture repair and the number of inpatient visits by physical and occupational therapists and related information. We will also check for any falls that participants may have while in hospital. In addition, we will record assessments of the four research areas (balance, bone health, cognition and continence) and physical therapy management that occurs as part of the B4 Clinic.

#### Descriptive Information

We will capture date of birth, sex, marital status, height and weight. Body mass index will be calculated as weight (kg)/height × meters^2^. The Functional Comorbidity Index will be used to estimate comorbidities associated with physical functioning [60]. We will determine pre-fracture function during the week prior to fracture; and will screen for depression [61], and monitor pain and fatigue [62].

### Quality of Life and Health Resource Utilization Measures

We will use the EQ-5D-5L, a generic health related quality of life measure [63]. This five question measure is easy to administer and can be used in cost-effectiveness studies to evaluate clinical interventions [63]. Respondents are asked to rate their health states for mobility, self-care, usual activities, pain/discomfort and anxiety/depression. We will use a capability measure, the ICECAP-O [64] to determine the effect of the intervention on participants' quality of life. We will also use a modified health resource utilization questionnaire [65] to capture self-report direct and indirect costs.

### Process Evaluation

We will use a log to record all eligible participants and will document reasons for ineligibility or refusal to participate. Adherence to the B4 Clinic will be monitored over the course of the intervention, and adverse events monitoring will occur throughout the study. We will invite participants to complete an exit interview to examine satisfaction with components of the intervention. We will also explore barriers and enablers to participation in the Clinic services; and specifically we will examine reasons for low attendance. Finally we will complete open-ended qualitative interviews with clinical and research staff throughout the study to document our progress. Members of the research team will review the findings from these interviews and adjust research administrative procedures if necessary and appropriate.

### Statistical Analysis

#### Sample Size

We plan to recruit 130 participants over an 18-month period. Allowing for 15% loss to follow-up we will have 110 evaluable participants at 12 months. We have chosen to power the study to detect fairly large effect sizes as the proposed intervention represents an intensive use of scarce resources (geriatrician, physical therapists) and would need to demonstrate large effects to be considered for adoption by health funders. *Justification for sample size*: Given the anticipated presence of ceiling and/or floor effects in the SPPB we will compare the two groups, without adjusting using a Wilcoxon-Mann-Whitney (WMW) test. The WMW test can be characterized by the WMW odds ratio, OR_wmw _= π/(1-π) where π = Pr (Y_1 _< Y_2_) + 0.5 Pr (Y_1 _= Y_2_). Note, if there is no difference between the groups, π = 0.5 or equivalently, OR_wmw _= 1.0. If the OR_wmw _is 2.0, the odds are 2:1 that Y_1 _is less than Y_2, _i.e. π = 2/3. With 55 evaluable participants/group we will have >80% power to detect ORs of 2.0 or greater assuming α = 0.05 (2-sided) [66].

#### Primary analysis

All analyses will be intention-to-treat; there will be no interim analysis. SPPB at 12 months post randomization will be compared between the two groups using the Wilcoxon-Mann-Whitney test. Point and interval estimates for the difference in medians will be calculated.

#### Secondary analyses

All secondary analyses will be for descriptive purposes only. Point and interval estimates of the effect of the intervention on LEM scores at 6 and 12 months will be determined using a linear mixed model. The baseline LEM score will be included in the model as a covariate. Analyses for frailty, TUG and activity patterns as measured by CHAMPS and accelerometry will follow that of LEM. Types of management received and adherence to prescribed therapies will be described separately for each group. Analyses will be conducted at 6 months for descriptive purposes only.

## Discussion

We have adopted the Integrated Knowledge Translation model for this project. We recognize the need for post-discharge hip fracture management and have worked together with clinicians to develop this model. Our preliminary work included interviews with seniors to pilot our outcome measures and better understand barriers and enablers to taking part in exercise after hip fracture. In addition, our team includes knowledge users such as our partners within the local health authority, and clinicians who work daily with this population. We have previously shown effectiveness of our falls clinic model [67] and now extend this work with a higher risk population. We aim to test a delivery model that would be acceptable to both older adults, their caregivers and clinicians; and will complete an economic evaluation in parallel to determine operating costs and cost-effectiveness and cost-utility of the intervention.

## Competing interests

The authors declare that they have no competing interests.

## Authors' contributions

WLC, KMK, PB, PG, HAM, and MCA contributed to the research question development and drafting the study protocol. MB, RAB, SB, MGD, HMH, CL, EMM, and JSG contributed to the development of the study protocol and dissemination plan design. WLC and MCA were responsible for drafting this publication, with significant contribution from KMK and HAM. All team members provided input to this manuscript and approved the final version.

## Pre-publication history

The pre-publication history for this paper can be accessed here:

http://www.biomedcentral.com/1471-2318/11/30/prepub
